# Cardiovascular effects of dapagliflozin in patients with type 2 diabetes and different risk categories: a meta-analysis

**DOI:** 10.1186/s12933-016-0356-y

**Published:** 2016-02-19

**Authors:** Christian Sonesson, Peter A. Johansson, Eva Johnsson, Ingrid Gause-Nilsson

**Affiliations:** AstraZeneca Gothenburg, Pepparedsleden 1, SE-431 83 Mölndal, Sweden

**Keywords:** SGLT2 inhibitors, Cardiovascular, Type 2 diabetes, Dapagliflozin, Cardiovascular risk, Major adverse cardiovascular events, Meta-analysis

## Abstract

**Background:**

A pre-specified meta-analysis of cardiovascular (CV) events from 21 phase 2b/3 dapagliflozin clinical trials was undertaken to characterise the CV profile of dapagliflozin. This showed no increase in CV risk with dapagliflozin compared with control (placebo or comparator treatment) with or without background glucose-lowering therapies. The analysis reported here aimed to characterise the CV profile of dapagliflozin in subgroups of patients in these 21 studies grouped by degree of CV risk, based on both baseline and in-study risk factors (including hypoglycaemic events), with a focus on major adverse CV events (MACE).

**Methods:**

Patients with type 2 diabetes, both overall and with different levels of CV risk, including CV disease (CVD) history, age and other CV risk factors, were analysed. A further analysis compared CV risk in patients who experienced a hypoglycaemic event prior to MACE and those who did not. Analyses were based on time to first event using a Cox proportional hazards model stratified by study comparing dapagliflozin versus control.

**Results:**

In total, 9339 patients were included in this meta-analysis; 5936 patients received dapagliflozin 2.5–10 mg (6668 patient–years) and 3403 received control (3882 patient–years). Dapagliflozin is not associated with increased CV risk and results further suggest the potential for a beneficial effect both in the overall population [Hazard Ratio (HR) 0.77; 95 % CI (0.54, 1.10) for MACE] and in those with a history of CVD [HR 0.80 (0.53, 1.22)]. These findings were consistent in patients with varying degrees of CV risk, including age, number and type of CVD events in medical history and number of CV risk factors present. Furthermore, there was no increased risk of MACE in patients who experienced a hypoglycaemic event compared with those who did not.

**Conclusions:**

There was no suggestion of increased risk for MACE with dapagliflozin compared with control in any of the populations investigated. In addition, the results suggest the potential for a beneficial CV effect which is consistent with the multifactorial benefits on CV risk factors associated with sodium–glucose cotransporter-2 (SGLT2) inhibitors.

**Electronic supplementary material:**

The online version of this article (doi:10.1186/s12933-016-0356-y) contains supplementary material, which is available to authorized users.

## Background

In patients with type 2 diabetes mellitus (T2DM), cardiovascular disease (CVD) remains the leading cause of morbidity and mortality, with these individuals at approximately twice the risk of CVD compared with people without diabetes [1, 2]. Although rates have declined, a large burden still remains [3].

The link between improved glycaemic control and an improvement in microvascular outcomes is well established; however, although epidemiological evidence suggests a link [4–6], the effect on CVD risk is less clear [7–11]. In addition, the potential harm associated with severe hypoglycaemia might counterbalance the potential benefit of intensive glucose lowering treatment [12]. The need for personalised treatment of hyperglycaemia has been advocated [13–15] and a multifactorial approach to the treatment of risk factors is needed to decrease the cardiovascular (CV) risk [16, 17].

Dapagliflozin is a selective sodium–glucose cotransporter-2 (SGLT2) inhibitor that lowers blood glucose levels by reducing glucose reabsorption in the kidney independently of insulin secretion or action, resulting in increased urinary glucose excretion with associated osmotic diuresis and caloric loss [18]. The efficacy and safety of dapagliflozin has been studied in a wide range of populations as monotherapy or in combination with a variety of other glucose-lowering therapies [19–38]. The mechanism of action of dapagliflozin influences a number of CVD risk factors, in particular, decreasing blood pressure, reducing body weight (predominantly through reductions in total body fat mass, including visceral adipose tissue), reducing waist circumference, and lowering albuminuria and serum uric acid levels, with a low intrinsic risk of hypoglycaemia [26, 39, 40].

The US Food and Drug Administration (FDA) and European Medicines Agency (EMA) have issued guidance requiring that new diabetes therapies should rule out an unacceptable increase in CVD risk [41, 42]. Since then, a number of large CV outcome studies to clarify the effects of new classes of glucose-lowering therapies have either been conducted [43–47] or are ongoing [48–50]. Positive CV outcomes data, including a beneficial effect on hospitalisation for heart failure, were recently published for the SGLT2 inhibitor empagliflozin, which has a similar profile to dapagliflozin, showing the benefits of improving multiple CV risk factors with SGLT2 inhibitors [47]. Additionally, the ongoing large CV outcome trial DECLARE TIMI-58 [48] is being conducted in a broad population of patients with either established CVD or multiple CV risk factors and will aim to evaluate the effects of dapagliflozin on CV outcomes, with an estimated completion date of 2019.

In a pre-specified meta-analysis investigating CV outcomes in 21 trials from the dapagliflozin clinical development programme, which was conducted in line with the FDA guidance, no increase in CVD risk was observed in patients with T2DM receiving dapagliflozin compared with those receiving control (placebo or comparator treatment) with or without background glucose-lowering therapies [51]. The CV risk increases in patients with age, decrease in renal function and presence of one or several risk factors or comorbid conditions. The meta-analysis reported here aimed to characterise the CV profile of dapagliflozin overall and in subgroups of patients grouped by degree of CV risk, based both on baseline and in-study risk factors (i.e. hypoglycaemic events), with a focus on major adverse CV events (MACE).

## Methods

### Patient population

Data from Phase 2b studies of 12–24 weeks’ duration (five studies), and all Phase 3 studies of up to 208 weeks’ duration (16 studies) from the dapagliflozin clinical development programme in patients with T2DM, with the exception of an open-label Japanese study, were included (Additional file 1: Table S1). These studies compared dapagliflozin 2.5–10 mg with control (placebo or comparator treatment) as monotherapy or in combination with other glucose-lowering therapies, including metformin, sulfonylureas, thiazolidinediones, insulin and dipeptidyl peptidase 4 inhibitors. Methods for each individual study have previously been published [19–38]. All clinical study protocols were approved by the relevant institutional review boards/ethics committees and all enrolled patients provided written informed consent.

Analyses of CV outcomes were performed in several populations:

- In the overall population, comparison of CV outcomes according to the number of CV risk factors present in addition to T2DM. Cardiovascular risk factors included: age >65 years, history of CVD, history of hypertension, history of dyslipidaemia, smoking history, first degree family members with a history of premature coronary heart disease, and baseline estimated glomerular filtration rate (eGFR) <60 mL/min/1.73 m^2^.
- In a subpopulation with a history of CVD:
  - Comparison of CV outcomes according to the types of previous CVD events experienced and the number of different CVD event types in a patient’s history. The types of previous CVD events were defined as: coronary artery disease (myocardial infarction [MI], hospitalisation for unstable angina [UA], coronary artery bypass graft, percutaneous coronary intervention or stable angina), cerebrovascular disease (carotid artery disease, carotid endarterectomy or stenting, stroke or transient ischaemic attack), peripheral vascular disease (amputation, peripheral vascular disease or peripheral vascular surgery) or congestive heart failure (CHF).
- In a subpopulation of elderly patients aged ≥65 years with a history of both CVD and hypertension.
- Comparison of patients with or without hypoglycaemia prior to MACE in both the overall population and the subpopulation of patients with a history of CVD.

### Cardiovascular outcomes

Cardiovascular safety events were identified through an independent, blinded adjudication process, with standardised MedDRA (Medical Dictionary for Regulatory Activities) queries used to select the events for adjudication. In line with the FDA Guidance, the primary CVD event of interest in this meta-analysis was the composite of CVD death, MI, stroke and hospitalisation for UA (MACE plus UA) although we focus on MACE (composite of CVD death, MI and stroke) for the majority of the analyses here. Other CVD events investigated were the individual events of CV death, MI, stroke, hospitalisation for UA, unplanned coronary revascularisation, and hospitalisation for heart failure.

### Analysis methods

Analyses were based on time to first event using a Cox proportional hazards model stratified by study and including a term defining the treatment received by individual patients across the pooled studies (either dapagliflozin or control). Results were supported by Mantel–Haenszel methods (asymptotic and exact). Only studies with at least one adjudicated event contributed to the respective analyses. Any imbalances in CVD risk factors due to an unequal randomisation ratio were adjusted for in the analyses through stratification by study in the model. Hazard ratios (HRs) and 95 % confidence intervals (CIs) comparing dapagliflozin with control were calculated. An estimated HR <1 indicates a favourable effect of dapagliflozin versus control. Kaplan–Meier estimates for cumulative incidence were calculated for MACE + UA, MACE, their individual components (CV death, MI, stroke and UA) and hospitalisation for heart failure.

## Results

### Patients

Overall there were 9339 patients included in this meta-analysis, with 10,550 patient–years of exposure to study drug; 5936 patients received dapagliflozin (6668 patient–years) and 3403 received control (3882 patient–years). There were 3214 patients with a history of CVD (1856 and 1358 treated with dapagliflozin and control, respectively); the subgroup of elderly patients (≥65 years) with a history of CVD and hypertension included 1263 patients from 19 studies (707 patients treated with dapagliflozin and 556 with control). Patient demographics and baseline characteristics were balanced between dapagliflozin and control groups within each studied population (Table 1). Patients with history of CVD, and elderly patients with history of CVD and hypertension, were older, had a longer T2DM duration, more impaired renal function, lower low-density lipoprotein (LDL)-cholesterol and higher mean systolic blood pressure, compared with patients in the overall population. In the overall population, a slight imbalance in history of CVD, hypertension and CHF between dapagliflozin and control groups was observed and this was accounted for in the analysis.

**Table 1:** Demographics and baseline characteristics

	All patients		Patients with history of CVD		Elderly patients with hypertension and a history of CVD	
	DAPA (N = 5936)	CTRL (N = 3403)	DAPA (n = 1856)	CTRL (n = 1358)	DAPA (n = 707^a^)	CTRL (n = 556)
Age, mean (SD), years	56.9 (10.4)	58.1 (10.3)	62.4	62.9	70.2 (4.1)	70.1 (4.2)
≥65 years (%)	24.0	28.8	40.9	43.1	100	100
Race						
White, n (%)	4505 (75.9)	2644 (77.7)	1634 (88.0)	1199 (88.3)	634 (89.7)	521 (93.7)
Other^b^, n (%)	1431 (24.1)	759 (22.3)	222 (12.0)	159 (11.7)	73 (10.3)	35 (6.3)
BMI, mean (SD), kg/m^2^	31.3 (5.7)	31.6 (5.8)	32.4 (5.4)	32.5 (5.9)	31.8 (5.0)	32.1 (5.4)
T2DM duration, mean (SD), years	7.0 (7.5)	7.6 (7.7)^c^	11.1 (8.4)	11.2 (8.3)	13.4 (9.3)	13.0 (9.1)
History of CVD (%)	31.3	39.9	100	100	100	100
History of hypertension (%)	65.7	71.9	89.8	92.8	100	100
History of CHF (%)	3.9	4.8	12.6	12.0	15.4	15.3
Smoking history (%)	43.3	46.3	53.3	56.4	51.8	53.1
eGFR, mean (SD), mL/min/1.73 m^2^	83.9 (21.3)	83.6 (21.1)^d^	75.7 (19.5)	77.0 (19.5)^e^	69.9 (18.5)	71.7 (17.9)
LDL, mean (SD), mmol/L	2.78 (0.98)^f^	2.66 (0.96)^g^	2.47 (0.98)^h^	2.36 (0.92)^i^	2.32 (0.90)^j^	2.28 (0.89)^k^
SBP, mean (SD), mmHg	130.4 (15.7)^l^	131.1 (14.9)^m^	134.5 (15.4)^n^	133.7 (14.5)^o^	136.7 (15.7)^p^	135.6 (14.0)^q^
DBP, mean (SD), mmHg	78.8 (9.1)^l^	78.8 (8.9)^m^	78.0 (9.3)^n^	77.7 (9.1)^o^	76.1 (9.3)^p^	76.1 (9.0)^q^
Concomitant medications of interest:						
Any antihypertensive	3217 (54.2)	2061 (60.6)	1440 (86.4)	1085 (86.1)	604 (85.4)	476 (85.6)
Diuretics	1509 (25.4)	960 (28.2)	788 (47.3)	604 (47.9)	363 (51.3)	282 (50.7)
β-blockers	1604 (27.0)	1127 (33.1)	1045 (62.7)	819 (65.0)	454 (64.2)	367 (66.0)
ACEi/ARBs	2938 (49.5)	1908 (56.1)	1327 (79.7)	1022 (81.1)	556 (78.6)	451 (81.1)
Calcium channel blockers	1126 (19.0)	717 (21.1)	545 (32.7)	431 (34.2)	251 (35.5)	203 (36.5)
Statins	2276 (38.3)	1582 (46.5)	1145 (68.7)	928 (73.7)	497 (70.3)	408 (73.4)
Aspirin	1906 (32.1)	1322 (38.8)	1068 (64.1)	858 (68.1)	456 (64.5)	378 (68.0)

*ACEi* angiotensin converting enzyme inhibitors, *ARBS* angiotensin receptor blockers, *BMI* body mass index, *CHF* congestive heart failure, *CTRL* control, *CVD* cardiovascular disease, *DAPA* dapagliflozin, *DBP* diastolic blood pressure, *eGFR* estimated glomerular filtration rate, *LDL* low density lipoprotein cholesterol, *SD* standard deviation, *SBP* systolic blood pressure

^a^Two patients were not randomised to dapagliflozin, but were subsequently treated with dapagliflozin; ^b^ Other includes Black or African American, Asian and Other; ^c^ n = 3400; ^d^ n = 3402; ^e^ n = 1357; ^f^ n = 5742; ^g^ n = 3234; ^h^ n = 1821; ^i^ n = 1316; ^j^ n = 698; ^k^ n = 543; ^l^ n = 5619; ^m^ n = 3274; ^n^ n = 1824; ^o^ n = 1345; ^p^ n = 704; ^q^ n = 553

### Cardiovascular outcomes in the overall population

**Fig. 1:**
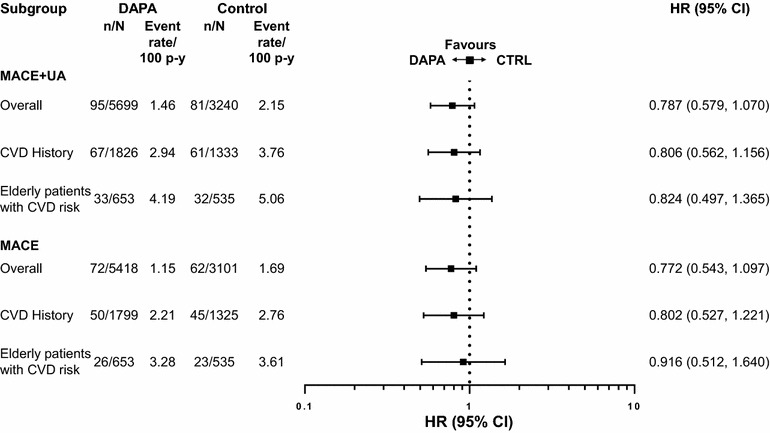
MACE + UA and MACE. Data presented for the overall population, the subgroup of patients with a history of CVD (CVD history) and the subgroup of elderly patients aged ≥65 years with a history of CVD and hypertension (Elderly patients with CVD risk). *n* is the number of patients with an event; *N* is the number of patients in treatment group. *CI* confidence interval, *CTRL* control, *CVD* cardiovascular disease, *DAPA* dapagliflozin, *HR* hazard ratio, *MACE* major adverse cardiovascular events (cardiovascular death, myocardial infarction and stroke), *MACE* + *UA* MACE plus unstable angina, p–y = patient years

**Fig. 2:**
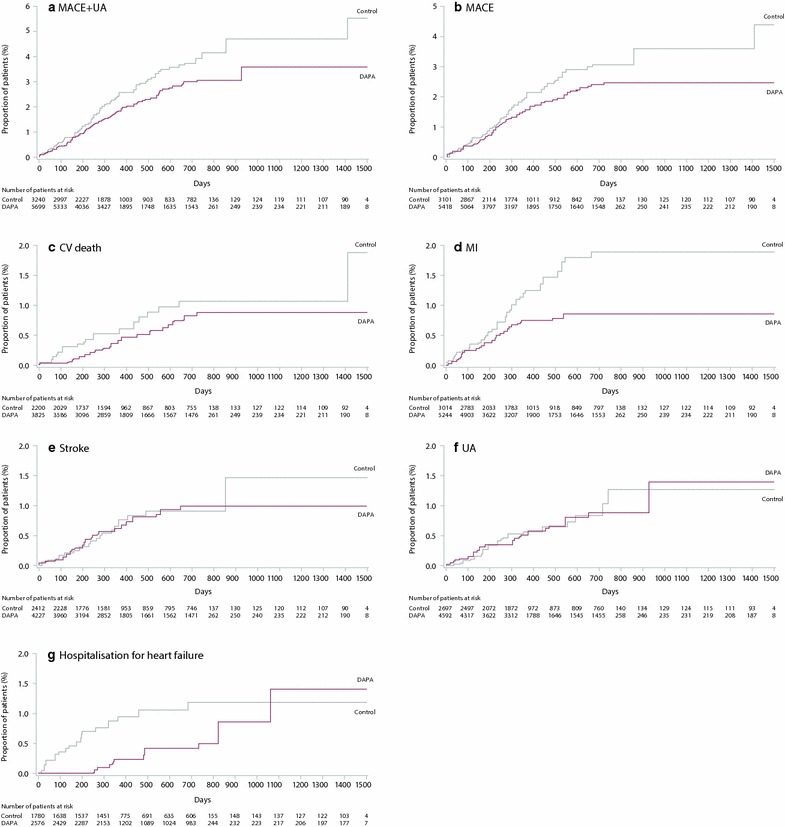
Cumulative incidence of **a** MACE + UA, **b** MACE, **c** CV death, **d** MI, **e** stroke, **f** UA and **g** hospitalisation for heart failure (Kaplan–Meier estimates). Data presented for the overall population. *CV* cardiovascular, *DAPA* dapagliflozin, *MACE* major adverse cardiovascular events (CV death, MI and stroke), *MI* myocardial infarction, *UA* unstable angina

**Fig. 3:**
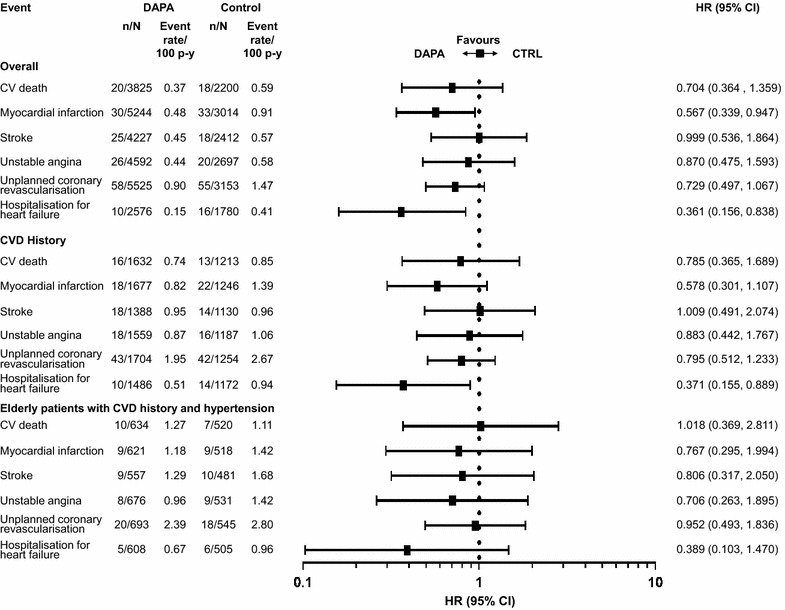
CV events. Data presented for the overall population, the subgroup of patients with a history of CVD (CVD history) and the subgroup of elderly patients aged ≥65 years with a history of CVD and hypertension (Elderly patients with CVD risk). *n* is the number of patients with an event; *N* is number of patients in treatment group. *CI* confidence interval, *CTRL* control, *CV* cardiovascular, *CVD* CV disease, *DAPA* dapagliflozin, *HR* hazard ratio, p–y = patient years

**Fig. 4:**
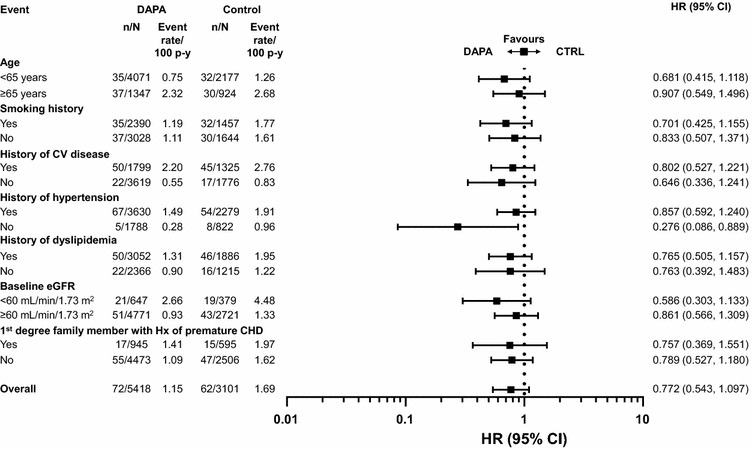
MACE in the overall population by presence or absence of CV risk factors. *n* is the number of patients with an event; *N* is the number of patients in treatment group. *CHD* coronary heart disease, *CI* confidence interval, *CV* cardiovascular, *CTRL* control, *DAPA* dapagliflozin, *eGFR* estimated glomerular filtration rate, *HR* hazard ratio, *Hx* history, *MACE* major adverse cardiovascular events (CV death, myocardial infarction and stroke), p–y = patient–years

**Fig. 5:**
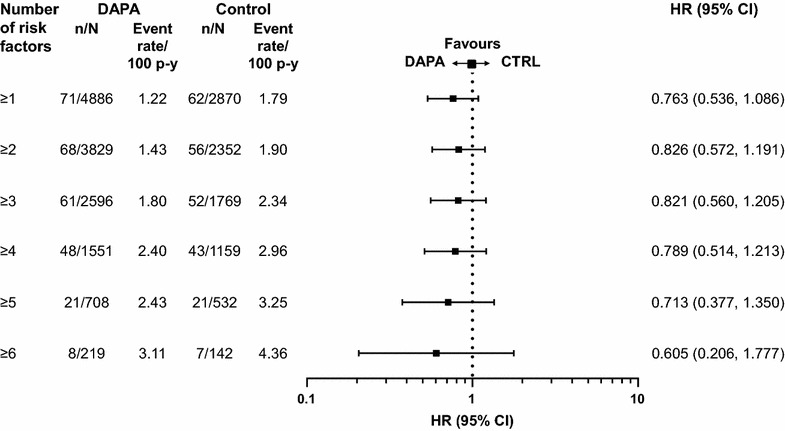
MACE in the overall population by number of CV risk factors present. *n* is the number of patients with an event; *N* is the number of patients in treatment group. *CI* confidence interval, *CTRL* control, *DAPA* dapagliflozin, *HR* hazard ratio, *MACE* major adverse cardiovascular events (CV death, myocardial infarction and stroke), p–y = patient–years

A total of 176 MACE plus UA events were observed in the overall population; 95 events in patients receiving dapagliflozin and 81 events in patients receiving control [HR 0.787; 95 % CI (0.579, 1.070)] (Fig. 1). A total of 134 MACE events (72 events in patients receiving dapagliflozin and 62 events in patients receiving control) were observed in the overall population [HR 0.772; 95 % CI (0.543, 1.097)] (Fig. 1). The cumulative probability of MACE + UA and MACE both showed a gradual separation of the dapagliflozin and control curves during the treatment period (Fig. 2). There was a consistent pattern, with beneficial or neutral point estimates for all individual types of CV events in dapagliflozin- compared with control-treated patients (Fig. 3), including a beneficial estimate on hospitalisation for heart failure [HR 0.361; 95 % CI (0.156, 0.838)] (Fig. 3), which showed an early separation of the cumulative probability of an event between the treatment groups (Fig. 2); albeit only based on 26 events. For all Kaplan–Meier plots in Fig. 2, the relatively few events occurring in the later time period should be noted. The presence or absence of specific CVD risk factors (including family history of premature coronary heart disease, baseline eGFR, dyslipidaemia, hypertension, smoking, history of CVD and older age), did not generally affect the estimated HRs, which were less than 1 in all subgroups analysed (Fig. 4). When patients were considered according to the present number of CVD risk factors, estimated HRs were less than 1 for all categories (≥1, ≥2, ≥3, ≥4, ≥5 or ≥6 risk factors) with a tendency towards higher event rates with increasing number of risk factors in both the dapagliflozin and the control groups (Fig. 5).

### Cardiovascular outcomes in patients with a history of CVD

A total of 128 MACE plus UA events were observed in the subgroup of patients with a history of CVD; 67 events in patients receiving dapagliflozin and 61 events in patients receiving control [HR 0.806; 95 % CI (0.562, 1.156)] (Fig. 1). A total of 95 MACE events were observed in this subgroup; 50 in patients receiving dapagliflozin and 45 in patients receiving control [HR 0.802; 95 % CI (0.527, 1.221)] (Fig. 1). The risk for MACE events with dapagliflozin compared with control in patients without a history of CVD also favoured dapagliflozin [HR 0.646; 95 % (0.336, 1.241)] (Fig. 4). The pattern of beneficial or neutral point estimates for all individual types of CV events in dapagliflozin- compared with control-treated patients and a beneficial point estimate for dapagliflozin versus control for hospitalisation for heart failure, was also seen in this population (Fig. 3). This was also true for MI, where similar results were observed in the overall population and patients with a history of CVD, albeit with fewer events in the latter population.

**Fig. 6:**
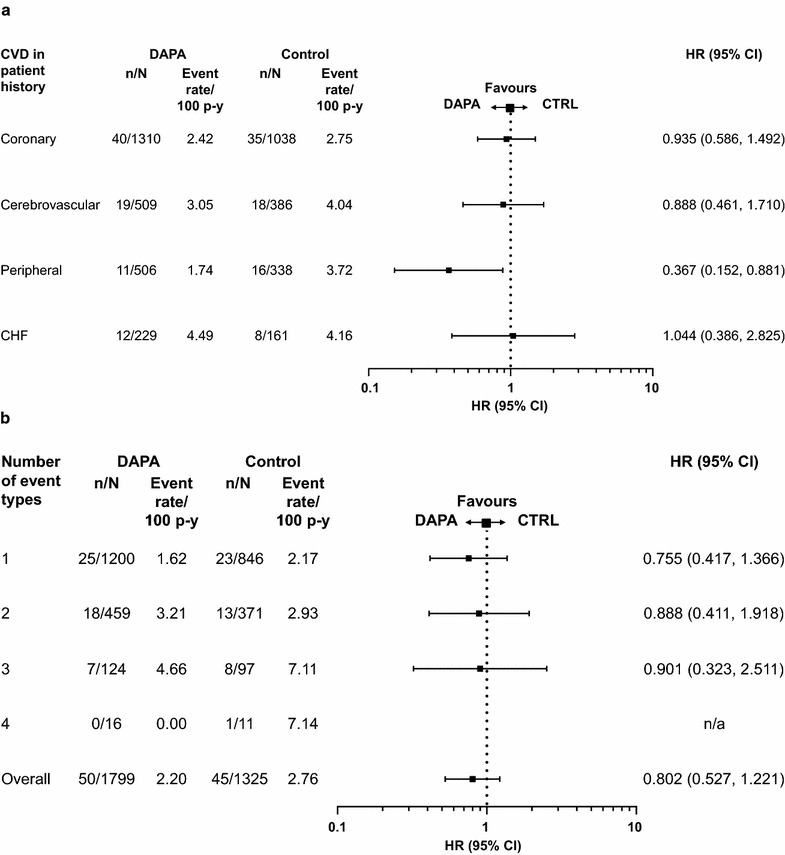
Risk of MACE in the subgroup of patients with a history of CVD. (CVD history). Data presented by **a** the different types of CVD in patient history, and **b** the number of CVD event types in patient history. *n* is the number of patients with an event; *N* is the number of patients in treatment group. Coronary = coronary artery disease (myocardial infarction, hospitalisation for unstable angina, coronary artery bypass graft, percutaneous coronary intervention or stable angina); cerebrovascular = cerebrovascular disease (carotid artery disease, carotid endarterectomy or stenting, stroke or transient ischaemic attack); peripheral = peripheral vascular disease (amputation, peripheral vascular disease or peripheral vascular surgery). *CHF* congestive heart failure, *CI* confidence interval, *CTRL* control, *CV* cardiovascular, *CVD* CV disease, *DAPA* dapagliflozin, *HR* hazard ratio, *MACE* major adverse cardiovascular events (CV death, myocardial infarction and stroke), *n/a* not available, p–y = patient–years

When patients were considered according to types of CVD history (Fig. 6a) or number of distinct CVD types experienced in their history (according to the same classes; Fig. 6b), there was no increase in risk for MACE in patients treated with dapagliflozin compared with those treated with control.

### Cardiovascular outcomes in elderly patients with a history of CVD and hypertension

In the subgroup of elderly patients aged at least 65 years who had a history of CVD and hypertension, a total of 65 MACE plus UA events were observed (33 in patients receiving dapagliflozin and 32 in patients receiving control) [HR 0.824; 95 % CI (0.497, 1.365)] (Fig. 1), and a total of 49 MACE events were observed (26 in patients receiving dapagliflozin and 23 in patients receiving control) [HR 0.916; 95 % CI (0.512, 1.640)]. There was no increase in risk for any of the individual components of MACE in dapagliflozin-treated patients compared with those receiving control, although the number of events was low for each individual endpoint (Fig. 3).

### Cardiovascular outcomes in patients with or without hypoglycaemia prior to MACE

**Fig. 7:**
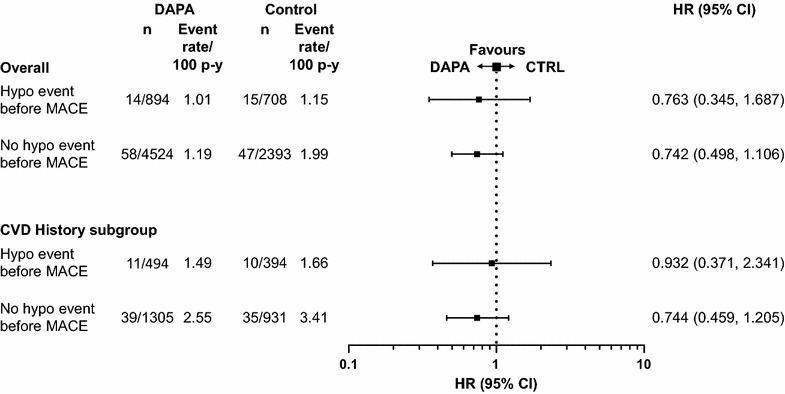
MACE by occurrence of hypoglycaemic event. Data presented for the overall population and the subgroup of patients with a history of CVD (CVD history). *n* is the number of patients with an event; *N* is the number of patients in treatment group. *CI* confidence interval, *CTRL* control, *CVD* cardiovascular disease, *DAPA* dapagliflozin, *HR* hazard ratio, *hypo* hypoglycaemia, *MACE* major adverse cardiovascular events (cardiovascular death, myocardial infarction and stroke), p–y = patient–years

No increased risk for MACE was observed with dapagliflozin compared with control in patients who did or did not experience a hypoglycaemic event prior to a first MACE event. This was consistently observed in both the overall population and the subpopulation of patients with a history of CVD (Fig. 7). The MACE event rates observed were not consistently higher in patients with a hypoglycaemic event, rather the opposite, although the comparison is hampered by both types of events being post-randomisation events.

## Discussion

To carefully characterise the properties of dapagliflozin, the meta-analysis described here included a broad population of patients with a particular focus on those with an elevated risk for CV events, using different subpopulations of varying risk. The point estimates of the HRs for MACE and MACE plus UA were similar and in favour of dapagliflozin in the overall population as well as for the sub-populations with higher CV risk. Glycaemic variability and episodes of severe hypoglycaemia have been suggested as predictors of adverse CV outcomes in patients with T2DM [7, 52–58]. Dapagliflozin reduces hyperglycaemia independently of insulin secretion or action, and as such, has a low intrinsic propensity for hypoglycaemia. In our analysis there was no increased risk for MACE in patients treated with dapagliflozin who did or did not experience a hypoglycaemic event prior to the first MACE event, although the number of events was small.

The results reported here are consistent with previously published meta-analyses of the effect of SGLT2 inhibitors on CV events. One analysis included pooled data from clinical trials of several members of the SGLT2 inhibitor class, evaluating MACE plus UA in 17,180 patients from 25 studies (14 dapagliflozin, one empagliflozin and 10 canagliflozin studies) [59]. This analysis found no evidence for increased CV risk, with the HR point estimate in favour of the SGLT2 inhibitor [HR 0.89; 95 % CI (0.70, 1.14)] [59]. Meta-analysis provides a method for aggregating and interpreting data from multiple sources and is, therefore, an important tool for examining rare CV events that occur in diabetes clinical trials [60]. Although CV events here were prospectively adjudicated by an independent committee, one should note the limitations in the heterogeneous nature of the study populations, the post hoc nature of some of the analyses, the relatively low numbers of events and that data are not generated in a prospectively designed CV outcomes trial.

In addition to reducing hyperglycaemia in T2DM, dapagliflozin may improve glycaemic control in patients with T1DM [61]. Furthermore, dapagliflozin is known to have beneficial effects on several important CV risk factors, and two recent studies in patients with inadequately controlled hypertension and T2DM have reported clinically meaningful improvements in blood pressure, body weight, and serum uric acid levels with dapagliflozin [62, 63]. Dapagliflozin is also associated with beneficial effects on albuminuria [64, 65]; collectively suggesting a favourable CV profile. It has also been postulated that the mechanisms underlying the CV benefits of SGLT2 inhibitors could be even more multidimensional and may involve changes in arterial stiffness, cardiac oxygen demand, oxidative stress as well as other potential effects on the sympathetic nervous system, ventricular function and remodelling that remain to be elucidated [40]. Indeed, a murine model of obesity and T2DM indicates that empagliflozin may improve CV injury and remodelling, vascular dysfunction, and cognitive decline [66]; in addition to reducing arterial stiffness in young patients with T1DM [67].

In line with this multifactorial risk factor hypothesis, and results generated from this and other meta-analyses that investigated the effects of SGLT2 inhibitors on CV outcomes, positive results were also recently reported with empagliflozin, in the first CV outcomes study with an SGLT2 inhibitor in patients with T2DM and established CVD [47]. Superiority for the primary outcome of MACE was driven by significantly lower rates of death from CV causes while there was no statistical difference between the treatment groups in rates of MI or stroke. Significantly lower rates of hospitalisation for heart failure and death from any cause were also observed; consistent with the current analysis, which reported beneficial or neutral point estimates for all CV events with dapagliflozin, including a beneficial estimate on hospitalisation for heart failure. It should be noted that eGFR levels were higher in the current study than the empagliflozin study [47] [baseline eGFR (SD): 83.9 (21.3) and 83.6 (21.1) mL/min/1.73 m^2^ with dapagliflozin and control, respectively; vs. 74.2 (21.6) and 73.8 (21.1) mL/min/1.73 m^2^ with empagliflozin and placebo], which could potentially impact on the overall frequency of CV events.

In the empagliflozin CV outcomes study, the cumulative probability of the primary outcome showed an early separation between the treatment groups [47]. In the current meta-analysis, a gradual separation was seen between the dapagliflozin and control curves, based on 134 MACE events. Variability may play a role in the different patterns observed in the current analysis and the empagliflozin outcome study, due to substantial differences in study design and population. Dapagliflozin and empagliflozin have similar profiles and no established mechanism suggests a different time to effect. Similar time to effect patterns were observed for dapagliflozin and empagliflozin for hospitalisation for heart failure, although as previously noted this was based on only 26 events in this meta-analysis.

The CV effects of SGLT2 inhibitors calls for further studies and confirmation. The prospective, randomised CV outcomes trial DECLARE TIMI-58 [48], with an estimated enrolment of 17,150 patients and expected median follow-up of more than 4 years, will document the effects of dapagliflozin on CV outcomes in patients ≥40 years old with T2DM and established CVD or multiple CV risk factors [48]. This study is uniquely positioned by its broader patient population, including both established CVD and multiple risk factor patients, large sample size and long-term follow-up to provide further evidence on the effects of SGLT2 inhibitors on CV risk.

## Conclusions

In this meta-analysis of data from across the dapagliflozin clinical development programme, including high CV risk patients, there was no evidence for increased risk of major adverse CV events with dapagliflozin. The results suggest the potential for a beneficial CV effect by dapagliflozin which is consistent with the multifactorial benefits on CV risk factors associated with SGLT2 inhibitors.

## Additional file

10.1186/s12933-016-0356-y Studies included in the meta-analysis.

## Abbreviations

CHF: congestive heart failure
CI: confidence interval
CV: cardiovascular
CVD: cardiovascular disease
DECLARE TIMI-58: dapagliflozin effect on cardiovascular events TIMI-58
eGFR: estimated glomerular filtration rate
EMA: European Medicines Agency
FDA: US Food and Drug Administration
HR: hazard ratio
LDL: low-density lipoprotein
MACE: major adverse cardiovascular events
MACE+UA: major adverse cardiovascular events + hospitalisation for unstable angina
MedDRA: Medical Dictionary for Regulatory Activities
MI: myocardial infarction
SGLT2: sodium–glucose cotransporter 2
T2DM: type 2 diabetes mellitus
UA: unstable angina
US: United States
