# Supplementary material for: Cardiovascular effects of dapagliflozin in patients with type 2 diabetes and different risk categories: a meta-analysis
Source: Cardiovasc Diabetol. 2016 Feb 19;15:37. doi: 10.1186/s12933-016-0356-y (PMC4761166; doi:10.1186/s12933-016-0356-y)
Supplement: Supplementary file 1 — 10.1186/s12933-016-0356-y Studies included in the meta-analysis. [file 12933_2016_356_MOESM1_ESM.docx]

**Table S1.** Studies included in the meta-analysis

| **Study** | **Description** | **Comparator** | **Dapagliflozin arm, N** | **Comparator arm, N** | **Duration (weeks)** |
| --- | --- | --- | --- | --- | --- |
| NCT00263276 [19] | Monotherapy | PBO, MET | 253 | 95 | 12 |
| NCT00357370 [20] | Add-on to insulin | PBO | 48 | 23 | 12 |
| NCT00528372 [33] | Monotherapy | PBO | 410 | 75 | 24 + 78 |
| NCT00528879 [21] | Add-on to MET | PBO | 409 | 137 | 24 + 78 |
| NCT00643851 [22] | Initial combination with MET | MET | 397 | 201 | 24 |
| NCT00660907 [23] | Add-on to MET | SU | 406 | 408 | 52 + 52 + 104 |
| NCT00680745 [24] | Add-on to SU | PBO | 450 | 146 | 24 + 24 |
| NCT00663260 [25] | Moderate renal impairment; add-on to usual care | PBO | 168 | 84 | 24 + 28 + 52 |
| NCT00683878 [26] | Add-on to TZD | PBO | 281 | 139 | 24 + 24 |
| NCT00736879 [34] | Low dose monotherapy | PBO | 142 | 68 | 24 |
| NCT00673231 [27] | Add-on to insulin | PBO | 610 | 197 | 24 + 24 + 56 |
| NCT00859898 [22] | Initial combination with MET | PBO, MET | 430 | 208 | 24 |
| NCT00976495 [35] | Effect on eGFR; add on to MET and/or SU | PBO, HCTZ | 24 | 51 | 12 |
| NCT00855166 [28] | Add-on to MET | PBO | 91 | 91 | 24 + 78 |
| NCT00831779 [36] | Insulin sensitivity; add on to MET ± insulin secretagogue | PBO | 23 | 21 | 12 |
| NCT01095653 [37] | Multinational Asia; monotherapy | PBO | 261 | 132 | 24 |
| NCT00984867 [29] | Add-on to SITA ± MET | PBO | 225 | 226 | 24 + 24 |
| NCT00972244 [38] | Japan; monotherapy | PBO | 166 | 54 | 12 |
| NCT01031680 [30] | High CV risk; add-on to usual care | PBO | 460 | 462 | 24 + 28 + 52 |
| NCT01042977 [31] | High CV risk; add-on to usual care | PBO | 482 | 483 | 24 + 28 + 52 |
| NCT01294423 [32] | Japan; monotherapy | PBO | 174 | 87 | 24 |

CV=cardiovascular; eGFR=estimated glomerular filtration rate; HCTZ=hydrochlorothiazide; MET=metformin; PBO=placebo; SITA=sitagliptin; SU=sulfonylurea; TZD=thiazolidinedione
